# Safety of Roxadustat in Chronic Kidney Disease Patients: An Updated Systematic Review and Meta-Analysis

**DOI:** 10.3390/ph18101566

**Published:** 2025-10-17

**Authors:** Patricia Martínez-Miguel, Encarnación Fernández-Antón, Diego Rodríguez-Puyol, Francisco J. de Abajo, Susana López Ongil

**Affiliations:** 1 Servicio de Nefrología, Hospital Universitario Príncipe de Asturias, 28805 Alcalá de Henares, Madrid, Spain; pmartinez.hupa@salud.madrid.org (P.M.-M.); drodriguez.hupa@gmail.com (D.R.-P.); 2 Departamento de Ciencias Biomédicas, Facultad de Medicina y Ciencias de la Salud, Universidad de Alcalá (IRYCIS Area 6), 28871 Alcalá de Henares, Madrid, Spain; encarnacion.fernande@uah.es (E.F.-A.); francisco.abajo@uah.es (F.J.d.A.); 3 Area 6-Epidemiotogía, Salud Pública y Asistencia Sanitaria del IRYCIS, 28046 Madrid, Spain; 4 Fundación Para la Investigación Biomédica, Hospital Universitario Príncipe de Asturias, 28805 Alcalá de Henares, Madrid, Spain; 5 Departamento de Medicina y Especialidades Médicas, Facultad de Medicina y Ciencias de la Salud, Universidad de Alcalá, 28805 Alcalá de Henares, Madrid, Spain; 6 Area 3-Fisiología y Fisiopatología Renal y Vascular del IRYCIS, 28046 Madrid, Spain; 7 Unidad de Farmacología Clínica, Hospital Universitario Príncipe de Asturias, 28805 Alcalá de Henares, Madrid, Spain

**Keywords:** roxadustat, chronic kidney disease, anemia, safety, dialysis

## Abstract

**Background/Objectives:** Roxadustat is a new treatment for the anemia of chronic kidney disease (CKD) that has comparable efficacy to erythropoietic-stimulating agents (ESAs), with the advantage of oral administration and increased iron bioavailability. It appears to be a safe treatment in terms of the development of major adverse cardiovascular events (MACEs); however, its long-term safety has not been fully evaluated. In this meta-analysis we evaluate its safety in dialysis-dependent (DD) and non-dialysis-dependent (NDD) CKD patients, considering the comparator used and treatment duration. **Methods:** The safety of Roxadustat was assessed based on the incidence of serious (SAEs) and non-serious adverse events (AEs). A random-effects method was used to estimate the odds ratios (ORs) and their 95% CIs. **Results:** Fifteen different randomized controlled clinical trials were included, with a total of 10,284 patients with CKD stages 3–5 treated with Roxadustat, 5604 on dialysis and 4680 not on dialysis. The overall incidence of AEs in the Roxadustat group did not change significantly (OR = 1.13; 1.00–1.27); however, the incidence of SAEs was significantly higher than in the control group (OR = 1.13; 1.04–1.23). Specifically, the incidence of hypertension (OR = 1.39; 1.13–1.73) and hyperkalemia (OR = 1.31; 1.02–1.69) was higher in the Roxadustat group than in the placebo group of NDD patients. All AEs except MACEs and hyperkalemia increased with treatment > 30 weeks. No differences were found in the incidence of any adverse effects studied compared with ESAs. **Conclusions:** Roxadustat is associated with an increased risk of SAEs, including hypertension and hyperkalemia in NDD patients. Therefore, monitoring potassium levels and blood pressure is recommended in these patients.

## 1. Introduction

Renal anemia is a common complication of chronic kidney disease (CKD). The risk of anemia increases with the progression of kidney disease [1], affecting more than 90% of CKD patients on dialysis [2]. Anemia reduces the quality of life of dialysis patients and increases medical expenses, the incidence of cardiovascular events, hospitalization rates, and mortality [3,4]. There are many causes of renal anemia, including insufficient erythropoietin (EPO) production, reduced EPO activity, iron deficiency and other metabolic disorders, malnutrition, inflammatory states, massive blood loss, and more.

Currently, the main approach to correct anemia associated with CKD is the use of injectable erythropoiesis-stimulating agents (ESAs), administered intravenously or subcutaneously, as well as supplemental oral or intravenous iron therapy [5]. From now on, we will use the term ESAs to encompass short-acting recombinant human erythropoietin (rHuEPO; epoetin) and intermediate-acting darbepoetin alfa, which are the first-line treatment, as they are EPO-like agents with characteristics that include good tolerance and ease of use. Although ESAs may improve quality of life, many studies in dialysis-dependent CKD patients have found that when the protocol goal is to achieve higher hemoglobin (Hb) levels, the mortality rate is higher when using high doses of ESAs [6]. Some meta-analyses also support the association between adverse events and the administration of ESAs at high doses [7,8], as well as an increased risk of all-cause mortality and hypertension, regardless of Hb levels [9]. The high cost and safety concerns [10,11] of recombinant ESAs can be an issue, although concomitant iron supplementation can be cost-effective to reduce the ESA dose. However, excessive iron supplementation may cause iron overload and may increase the risk of cardiovascular events and mortality in hemodialysis patients [12]. Therefore, there is an urgent need to develop a safe and effective drug to correct renal anemia, which can be used long-term without increasing the risk of cardiovascular events in patients with CKD.

Hypoxia-inducible factor (HIF) regulates the cellular response to hypoxia, which is caused by an imbalance between oxygen supply and consumption and can lead to serious ischemic disorders, including cardiovascular disease. HIF is composed of two subunits: an oxygen-sensing HIF-α subunit and a constitutively expressed HIF-β subunit. Nangaku et al. identified novel HIF stabilizers as targeted molecular therapies against cardiovascular diseases [13]. HIF prolyl hydroxylase inhibitors are recently developed small molecule oral drugs for the treatment of renal anemia that act by stabilizing HIF. Among them, Roxadustat (FG-4592) has been the first in obtaining approval in the European Union for the treatment of anemia in both dialysis-dependent (DD) and non-dialysis-dependent (NDD) CKD patients. Prolyl hydroxylases (PHs) are responsible for the degradation of HIF under normal oxygen conditions, and Roxadustat binds to HIF-PHs, reversibly inhibiting them. Inhibition of these enzymes reduces HIF degradation, thus promoting its transcriptional activity by stabilizing HIF-α. This results in increased endogenous erythropoietin production, heme biosynthesis enzymes, and proteins that promote iron absorption and transport, thereby increasing erythropoiesis [13]. In patients with CKD anemia not requiring dialysis, Roxadustat elevates Hb levels in a dose-dependent manner, significantly decreases ferritin levels, and briefly increases endogenous EPO levels within or near the physiological range, compared to placebo at high doses. Studies indicate that Roxadustat was more effective than placebo and similar to ESAs in correcting anemia. An additional advantage is that it increases the bioavailability of iron and thus avoids the use of intravenous iron, as orally administered iron reduces iron metabolism disorders by elevating serum transferrin, improving intestinal iron absorption, and releasing stored iron [14,15]. In a phase 3 clinical trial, the efficacy and safety of Roxadustat was favorable compared to placebo and other ESAs, leading to its approval for the treatment of anemia in CKD on or off dialysis in Chile, China, the European Union, Japan, and South Korea [16,17,18,19].

Some studies combine CKD patients on dialysis with those not receiving dialysis, while others only study patients on or without dialysis. This meta-analysis of randomized controlled trials in CKD patients on or off dialysis aims to evaluate the safety of oral Roxadustat treatment for anemia, analyzing different subgroups based on the duration of Roxadustat treatment, whether the population is on dialysis, and the type of control used as a comparator (placebo or ESA). This research may provide further evidence on the safety of this new drug for the treatment of anemia in patients with CKD.

## 2. Materials and Methods

This systematic review and meta-analysis was conducted in accordance with the Guidelines for Review and Meta-Analysis (PRISMA) statement [20]. The review protocol has not been registered.

### 2.1. Data Sources and Search Strategies

We conducted a comprehensive review of relevant clinical trials published through September 2025 that report on the safety of Roxadustat, comparing different treatment durations in patients with CKD. The search was conducted using the PubMed search engines, the Embase database, and the Cochrane Central Register of Controlled Trials, limited to English-language studies. The search terms used were Roxadustat; anemia; chronic kidney disease; dialysis; safety; cardiovascular events; and adverse events. In addition, references of selected trials as well as relevant previous meta-analyses were examined to detect other eligible trials not identified by the automatic search. Duplicate studies were excluded.

### 2.2. Selection and Data Collection Process

Each search result was independently reviewed by two authors (E.F.-A. and S.L.O.) to determine eligibility. Any differences of opinion were resolved through discussion and consensus. If consensus was not reached, a third reviewer (F.J.d.A.) served as a referee.

### 2.3. Inclusion Criteria and Data Extraction

**Figure 1:**
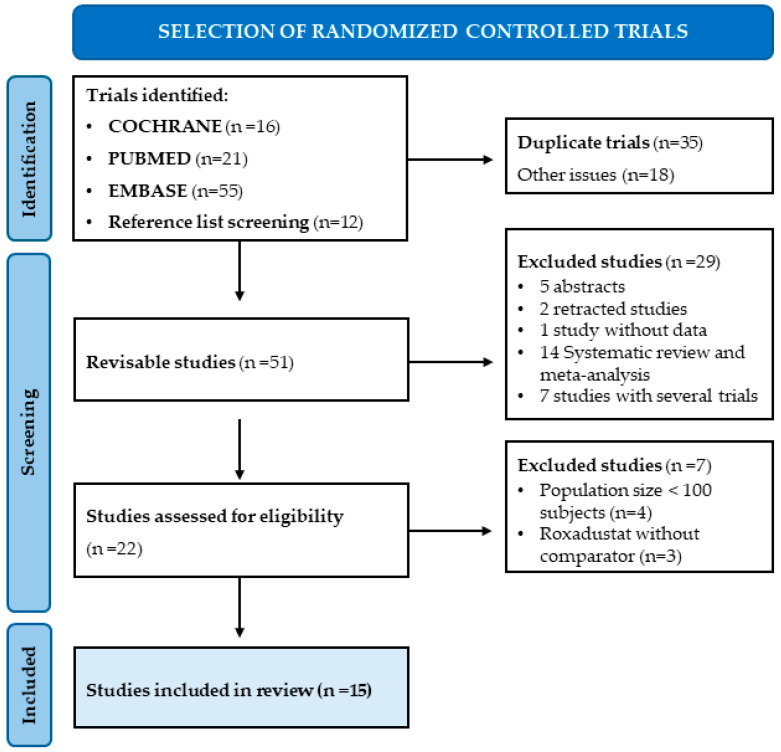
Flowchart of the study selection process.

In this systematic review and meta-analysis, we included clinical trials that met the following PICOS criteria: (1) Population: adults > 18 years with CKD and anemia receiving or not dialysis. (2) Intervention: exposure to oral Roxadustat. (3) Comparator: placebo or an ESA that could be rHuEPO, epoetin alpha, or darbepoetin alpha. (4) Outcome: the safety of Roxadustat, including different serious and non-serious adverse events in general, as well as the most common and important events occurring in CKD patients such as hypertension, diarrhea, hyperkalemia, and cardiovascular events. (5) Study: randomized controlled clinical trials with a sample size of at least 100 subjects overall. Finally, 15 trials met the inclusion criteria through full-text screening and were included in the systematic review and meta-analysis (Figure 1).

### 2.4. Data Items

The outcomes collected from each study were (1) duration of treatment with Roxadustat, (2) type of control, placebo or ESA, (3) whether or not patients were receiving dialysis, and (4) incidence of serious or non-serious adverse events, including cardiovascular events (MACEs), hypertension, diarrhea, and hyperkalemia, to assess the safety of Roxadustat.

### 2.5. Study Risk of Bias Assessment

The risk of bias of the included studies was assessed using the version 2 (August 2019) of the Cochrane risk-of-bias tool (RoB 2) for randomized controlled trials, evaluating five domains: randomization process, deviations from intended interventions, missing outcome data, outcome measurement, and selection of reported outcome.

### 2.6. Statistical Analysis

The odds ratio (OR) and its respective 95% confidence interval (CI) were calculated from the number of exposed and unexposed cases and the total number of treated patients given in each study, using a random-effects model. Results were shown as forest plots. Heterogeneity between trials was assessed using the Cochrane Q test (*p* < 0.10 indicates heterogeneity) and was reported as I-square (I^2^), which quantifies the variability between studies as a percentage. Heterogeneity was classified as low (I^2^ ≤ 40%), moderate (I^2^ ≤ 70%), or high (I^2^ > 70%). Heterogeneity was explored through subgroup analysis based on patient type (CKD patients with DD or NDD), type of comparator control (placebo or ESA) and duration of treatment with Roxadustat (≤30 or >30 weeks). In addition, potential publication bias in each analysis was quantitatively assessed by the correlation method between the effect measure and its precision described by Begg and Mazumdar [21], and by assessing the symmetry of the published data represented in a funnel plot. Publication bias was excluded if the correlation gave a *p* > 0.10. To further check the consistency of the results, a sensitivity analysis was performed excluding open-label trials.

All statistical analyses were performed with STATA/MP version 18 software.

## 3. Results

### 3.1. Characteristics of Included Trials

The initial automatic search in PubMed, the Cochrane Library and Embase identified 92 articles, of which 35 were duplicates and 18 addressed different topics. An additional 12 articles were identified by reviewing reference lists. Of the remaining 51 articles, 29 were excluded for various reasons: five studies were excluded after reviewing the title or abstract, two studies were retracted, one article lacked available data, fourteen were systematic reviews or meta-analyses, and seven included multiple trials. The remaining 22 potential trials were screened, of which 4 were excluded after full-text screening due to a population size of less than 100 subjects, and 3 were excluded because they used roxadustat without a comparator. Finally, 15 trials met all the inclusion criteria set for this systematic review and meta-analysis [14,16,17,18,22,23,24,25,26,27,28,29,30,31,32]. Figure 1 illustrates the flowchart of the study selection process. Eight of these trials were conducted with patients receiving dialysis and seven with patients not receiving dialysis. The characteristics of the included trials are described in Table 1. The included trials were published between 2015 and September 2025, and the sample size in each trial ranged from 100 to 2761. A total of 10,284 patients were enrolled. The duration of Roxadustat treatment ranged from 4 weeks to up to 4 years. The ages of the patients ranged from 18 to 80 years.

### 3.2. Safety Results

#### 3.2.1. Incidence of Adverse Events (AEs)

**Figure 2:**
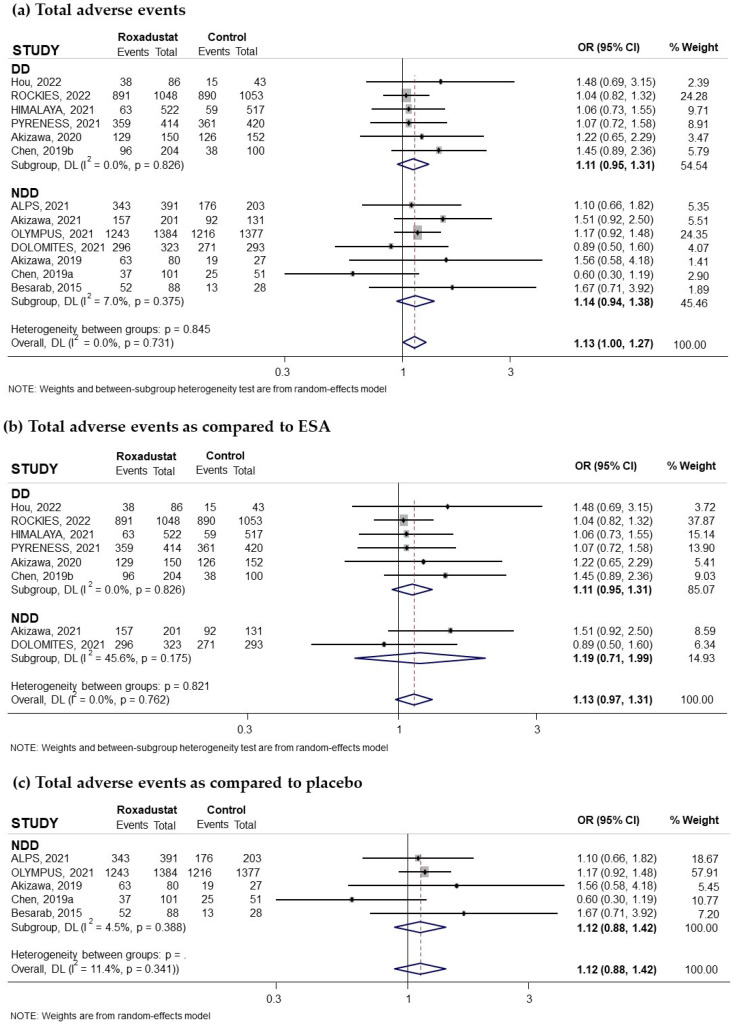
Incidence of adverse events by type of comparator [14,16,17,18,23,24,25,26,27,28,29,31,32]. Forest plot of the effect of Roxadustat on the incidence of adverse events in anemic patients with CKD depending on whether they are on dialysis or not, according to the type of comparator. (**a**) Patients with adverse events by study population, dialysis-dependent (DD) or non-dialysis-dependent (NDD) patients, comparing Roxadustat with the control group. Patients with adverse events analyzed by subgroups according to comparator, ESA (**b**) or placebo (**c**), within each population (DD or NDD). All results are presented as odds ratios (ORs) for treatment versus comparator, with their 95% confidence intervals (95% CIs).

**Figure 3:**
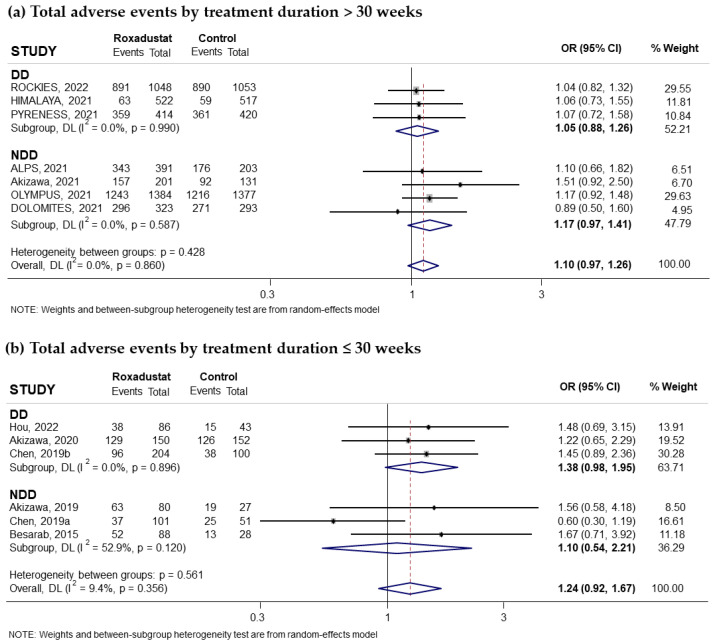
Incidence of adverse events by duration of treatment with Roxadustat [14,16,17,18,23,24,25,26,27,28,29,31,32]. Forest plot of the effect of Roxadustat on the incidence of adverse events in anemic patients with CKD depending on whether they are on dialysis or not, according to the duration of treatment with Roxadustat. Patients with adverse events analyzed according to the duration of treatment with Roxadustat, >30 weeks (**a**) or ≤30 weeks (**b**), within each population, dialysis-dependent (DD) or non-dialysis-dependent (NDD) patients. All results are presented as odds ratios (ORs) for treatment versus comparator, with their 95% confidence intervals (95% CIs).

Thirteen trials reporting the risk of adverse events related to oral Roxadustat treatment for anemia in patients with CKD were included in our study, and these events were collected when they affected at least 5% of subjects treated with Roxadustat. Seven of these trials had recruited non-dialysis-dependent patients and six had recruited patients on dialysis. The pooled odds ratio (OR) was 1.13 (95% confidence interval (CI): 1.00–1.27; I^2^ = 0.0%) (Figure 2a), indicating an increased risk bordering on significance, with no relevant differences observed between patients on dialysis (pooled OR = 1.11; 95% CI: 0.95–1.31; I^2^ = 0.0%) and not on dialysis (pooled OR = 1.14; 95% CI: 0.94–1.38; I^2^ = 7%) (Figure 2a). When Roxadustat was compared to ESAs, the pooled OR was 1.13 (95% CI: 0.97–1.31; I^2^ = 0.0%) (Figure 2b) and when compared to placebo, the pooled OR was 1.12 (95% CI: 0.88–1.42; I^2^ = 11.4%) (Figure 2c). Analysis according to the duration of treatment with Roxadustat showed no differences, finding a pooled OR of 1.10 (95% CI: 0.97–1.26; I^2^ = 0.0%) (Figure 3a) when the treatment was longer than 30 weeks and 1.24 (95% CI: 0.92–1.67); I^2^ = 9.4%) when it was less than 30 weeks (Figure 3b). No evidence of publication bias was observed according to the symmetry observed in the funnel plot and the correlation calculated by the Begg and Mazumdar test, *p* = 0.464 (Figure S1a).

#### 3.2.2. Incidence of Serious Adverse Events (SAEs)

**Figure 4:**
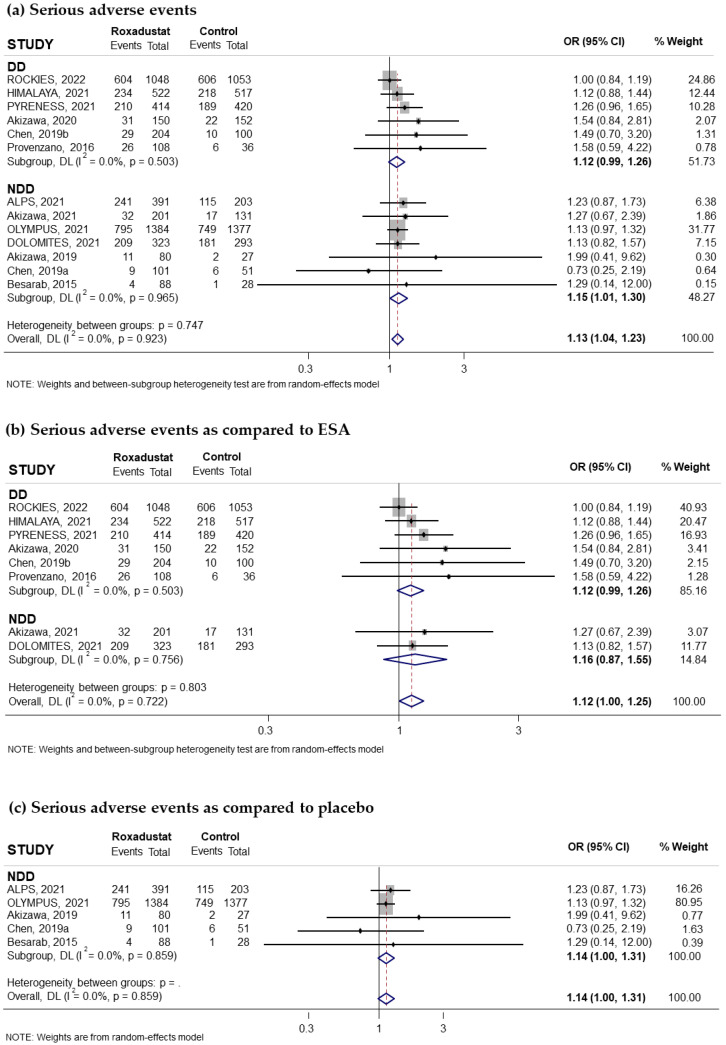
Incidence of serious adverse events by type of comparator [14,16,17,18,22,23,24,25,26,27,28,29,31]. Forest plot of the effect of Roxadustat on the incidence of serious adverse events in anemic patients with CKD depending on whether they are on dialysis or not, according to the type of comparator. (**a**) Patients with serious adverse events by study population, dialysis-dependent (DD) or non-dialysis-dependent (NDD) patients, comparing Roxadustat with the control group. Patients with serious adverse events analyzed by subgroups according to comparator, ESA (**b**) or placebo (**c**), within each population (DD or NDD). All results are reported as odds ratios (ORs) for treatment versus comparator, with their 95% confidence intervals (95% CIs).

Thirteen trials reported on the risk of SAEs associated with the use of oral Roxadustat; these events were collected when they affected at least 1% of subjects treated with Roxadustat. Seven of these trials had recruited non-dialysis-dependent patients and six had recruited patients on dialysis. The pooled OR was 1.13; (95% CI: 1.04–1.23; I^2^ = 0.0%) (Figure 4a). Subgroup analysis by dialysis or non-dialysis status indicated that the risk of SAEs was statistically significant in the anemic CKD patient group not receiving dialysis (pooled OR = 1.15; 95% CI: 1.01–1.30; I^2^ = 0.0%) (Figure 4a), while it was marginally non-significant in the dialysis patient group (pooled OR = 1.12; 95% CI: 0.99–1.26; I^2^ = 0.0%) (Figure 4a).

**Figure 5:**
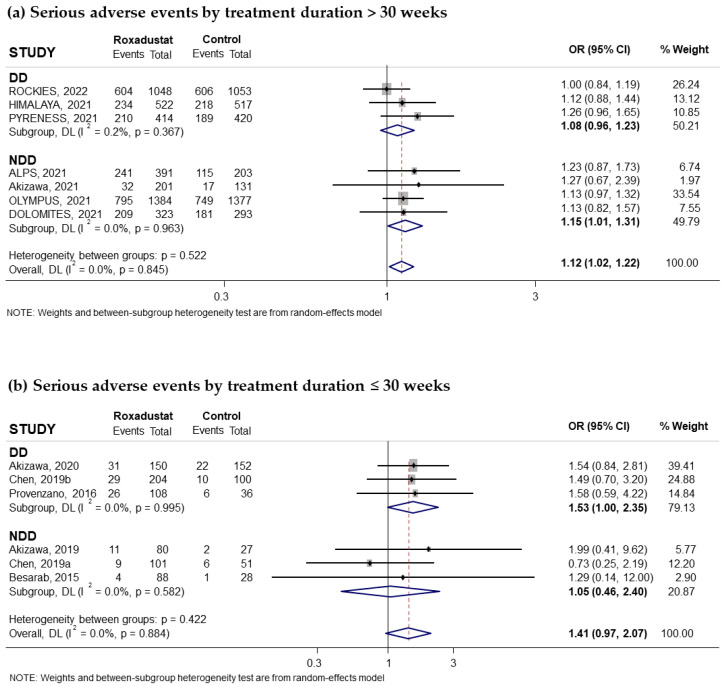
Incidence of serious adverse events by duration of treatment with Roxadustat [14,16,17,18,22,24,25,26,27,28,29,31]. Forest plot of the effect of Roxadustat on the incidence of serious adverse events in anemic patients with CKD depending on whether they are on dialysis or not, according to the duration of treatment with Roxadustat. Patients with serious adverse events analyzed according to the duration of treatment with Roxadustat, >30 weeks (**a**) or ≤30 weeks (**b**), within each population, dialysis-dependent (DD) or non-dialysis-dependent (NDD) patients. All results are reported as odds ratios (ORs) for treatment versus comparator, with their 95% confidence intervals (95% CIs).

According to the comparator used, the pooled OR was 1.12; (95% CI: 1.00–1.25; I^2^ = 0.0%) (Figure 4b) when compared to ESAs and 1.14 (95% CI: 1.00–1.31; I^2^ = 0.0%) when compared to placebo (Figure 4c). When analyzed by duration of treatment with Roxadustat, we found a pooled OR = 1.15 (95% CI: 1.01–1.31; I^2^ = 0.0%) for durations longer than 30 weeks (Figure 5a) in patients not receiving dialysis and 1.41 (95% CI: 0.97–2.07; I^2^ = 0.0%) if treatment was less than 30 weeks (Figure 5b). An association was observed when Roxadustat was used for more than 30 weeks in non-dialysis patients, pooled OR = 1.15 (95% CI: 1.01–1.31; I^2^ = 0.0%) (Figure S2a), but there was no association in dialysis patients, pooled OR = 1.08 (95% CI: 0.96–1.23; I^2^ = 0.2%) (Figure S2b). No publication bias was detected by the correlation calculated by the Begg–Mazumdar test, *p* = 0.272, greater than 0.05, although the funnel plot showed a slight asymmetry (Figure S1b).

### 3.3. Adverse Events

The most common adverse events were hypertension, diarrhea, hyperkalemia, and major adverse cardiovascular events (MACEs) in general, and are described below. No adverse events related to muscle spasms or respiratory infections were detected.

#### 3.3.1. Effect of Roxadustat on MACEs

**Figure 6:**
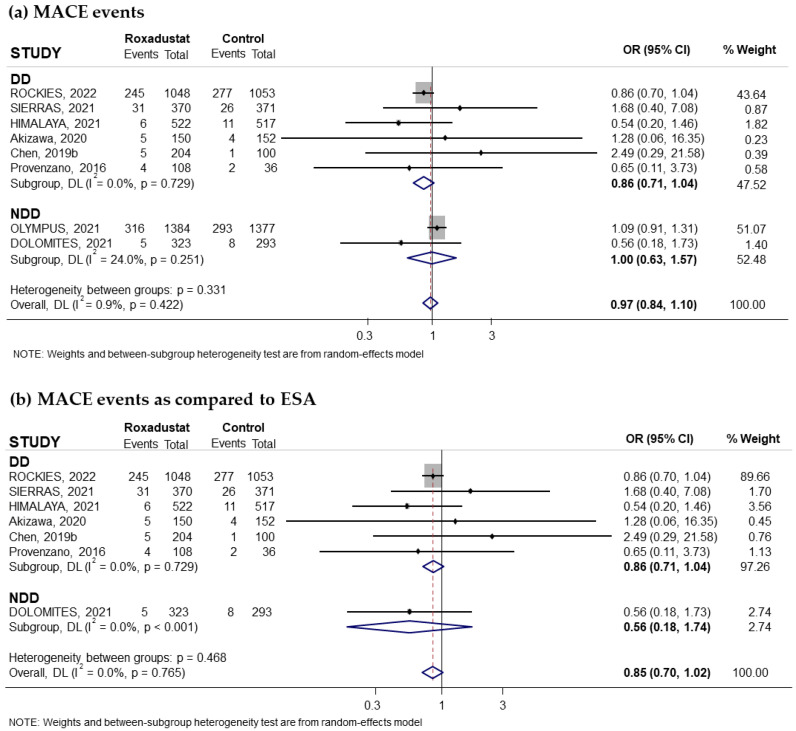
Incidence of MACEs by type of comparator [17,18,22,25,27,28,30,31]. Forest plot of the effect of Roxadustat on MACEs in anemic patients with CKD depending on whether they are on dialysis or not, according to the type of comparator. Patients with MACEs by study population, dialysis-dependent (DD) or non-dialysis-dependent (NDD) patients, comparing Roxadustat with the control group (**a**), and comparing Roxadustat with ESAs (**b**). All results are presented as odds ratios (ORs) for treatment versus comparator, with their 95% confidence intervals (95% CIs).

**Figure 7:**
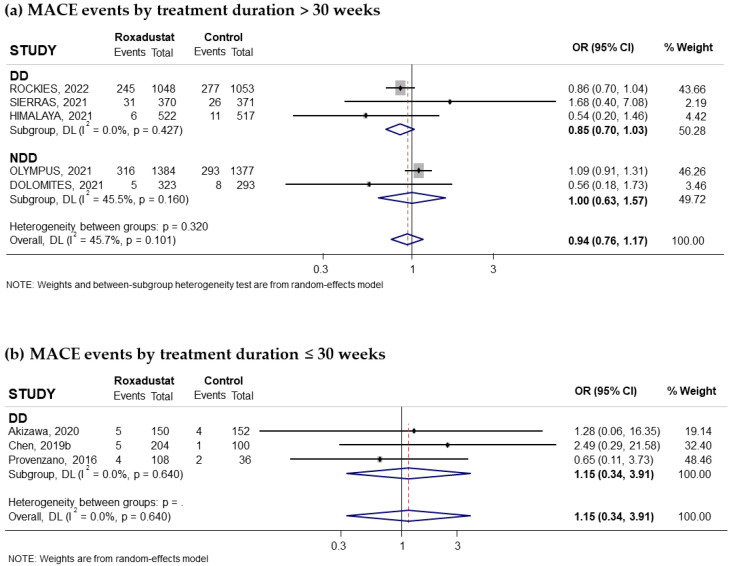
Incidence of MACEs by duration of treatment with Roxadustat [17,18,22,25,27,28,30,31]. Forest plot of the effect of Roxadustat on MACEs in anemic patients with CKD depending on whether they are on dialysis or not, according to the duration of treatment with Roxadustat. Patients with MACEs analyzed according to the duration of treatment with Roxadustat, >30 weeks (**a**) or ≤30 weeks (**b**), within each population, dialysis-dependent (DD) or non-dialysis-dependent (NDD) patients. All results are presented as odds ratios (ORs) for treatment versus comparator, with their 95% confidence intervals (95% CIs).

Eight trials reported on the risk of MACEs associated with oral Roxadustat treatment for anemia in CKD patients. Two of these trials had recruited non-dialysis-dependent patients and six had recruited patients on dialysis. The pooled OR was 0.97; 95% CI: 0.84–1.10; I^2^ = 0.9%) (Figure 6a). Subgroup analysis based on the comparator used did not reflect an increased risk of MACEs either compared to ESAs (Figure 6b). The results even show that Roxadustat is on the border of being significantly more beneficial than ESAs (pooled OR = 0.85; 95% CI: 0.70 to 1.02; I^2^ = 0.0%) (Figure 6b). Similarly, when analyzing the data by duration of Roxadustat treatment, no significant increased risk was observed either with longer (Figure 7a) or shorter (Figure 7b) treatment durations. Likewise, no significant changes were observed in either patients not on dialysis (Figure S3a) or patients on dialysis (Figure S3b) when Roxadustat treatment lasted longer than 30 weeks. The funnel plot, depicted in Figure S1c, was symmetric, suggesting absence of publication bias. A Begg and Mazumdar correlation test was not performed as there were only eight studies.

#### 3.3.2. Effect of Roxadustat on Hypertension

**Figure 8:**
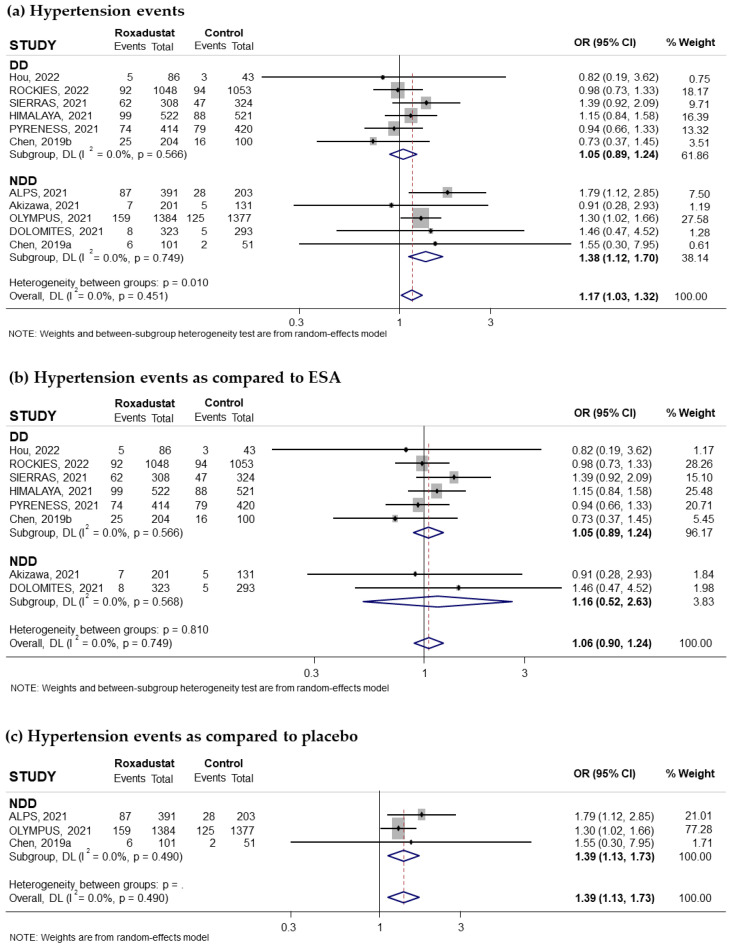
Incidence of hypertension events by type of comparator [16,17,24,25,26,27,28,29,30,31,32]. Forest plot of the effect of Roxadustat on hypertension events in anemic patients with CKD depending on whether they are on dialysis or not, according to the type of comparator. (**a**) Patients with hypertension events by study population, dialysis-dependent (DD) or non-dialysis-dependent (NDD) patients, comparing Roxadustat with the control group. Patients with hypertension events analyzed by subgroups according to comparator, ESA (**b**) or placebo (**c**), within each population (DD or NDD). All results are reported as odds ratios (ORs) for treatment versus comparator, with their 95% confidence intervals (95% CIs).

**Figure 9:**
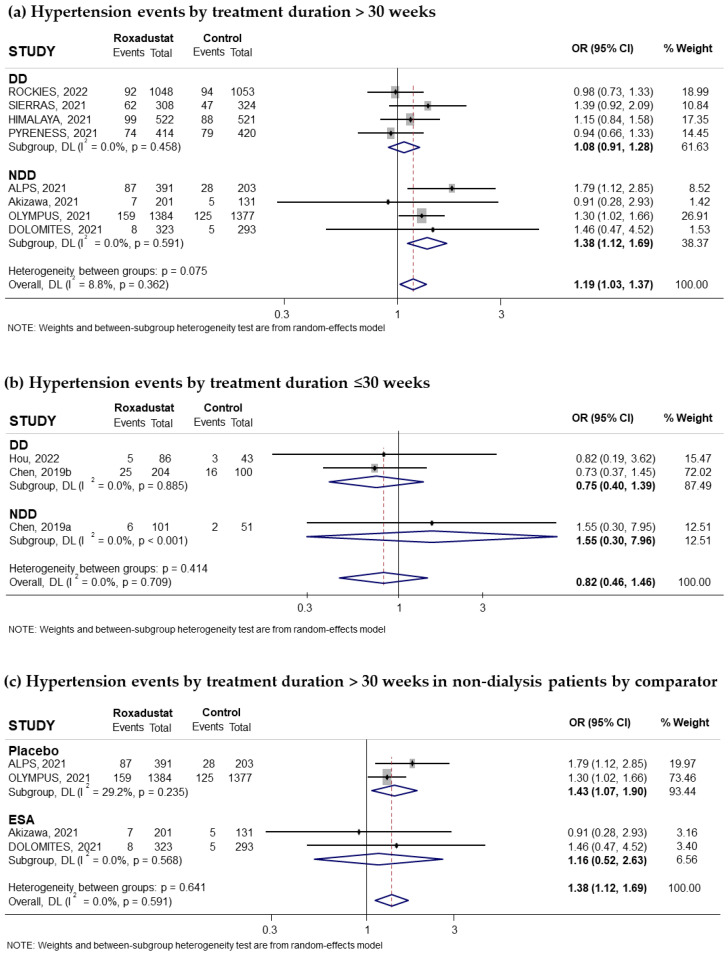
Incidence of hypertension events by duration of treatment with Roxadustat [16,17,24,25,26,27,28,29,30,31,32]. Forest plot of the effect of Roxadustat on hypertension events in anemic patients with CKD depending on whether they are on dialysis or not, according to the duration of treatment with Roxadustat. Patients with hypertension events analyzed according to the duration of treatment with Roxadustat, >30 weeks (**a**) or ≤30 weeks (**b**), within each population, dialysis-dependent (DD) or non-dialysis-dependent (NDD) patients. (**c**) Patients with hypertension events analyzed in NDD patients with treatment >30 weeks according to the type of comparator, ESA or placebo. All results are reported as odds ratios (ORs) for treatment versus comparator, with their 95% confidence intervals (95% CIs).

Eleven trials reporting the risk of hypertension associated with oral Roxadustat treatment for anemia in CKD patients were included. Five of these trials had recruited non-dialysis-dependent patients and six had recruited dialysis patients. The pooled OR was 1.17 (95% CI: 1.03–1.32; I^2^ = 0%) (Figure 8a). Subgroup analysis according to whether or not they receive dialysis suggested that the risk of hypertension was significant in non-dialysis-dependent patients (pooled OR = 1.38; 95% CI: 1.12–1.70; I^2^ = 0.0%) (Figure 8a), while it was lower and not significant in the group of patients on dialysis (pooled OR = 1.05; 95% CI: 0.89–1.24; I^2^ = 0.0%) (Figure 8a). According to the comparator used, the results showed a statistically significant increased risk when compared to placebo (pooled OR was 1.39; 95% CI: 1.13–1.73; I^2^ = 0.0%) (Figure 8c) but not when compared to ESAs (pooled OR was 1.06; 95% CI: 0.90–1.24; I^2^ = 0.0%) (Figure 8b). Regarding the duration of treatment, we found an increased risk of hypertension when the duration was longer than 30 weeks in patients not receiving dialysis (pooled OR = 1.38; 95% CI: 1.12–1.69; I^2^ = 0.0%) (Figure 9a) but not when the duration was less than 30 weeks (pooled OR = 0.82; 95% CI: 0.46–1.46; I^2^ = 0.0%) (Figure 9b).

Finally, a subgroup of non-dialysis patients receiving treatment for more than 30 weeks suggested that the risk of hypertension was significant when Roxadustat was compared to placebo (pooled OR was 1.43; 95% CI: 1.07–1.90; I^2^ = 29.2%) (Figure 9c). However, this association was not observed in non-dialysis patients when Roxadustat was used for more than 30 weeks compared to ESAs (Figure 9c). No evidence of publication bias was observed, based on funnel plot and Begg–Mazumdar correlation, *p* = 0.697 (Figure S1d).

#### 3.3.3. Effect of Roxadustat on Diarrhea

**Figure 10:**
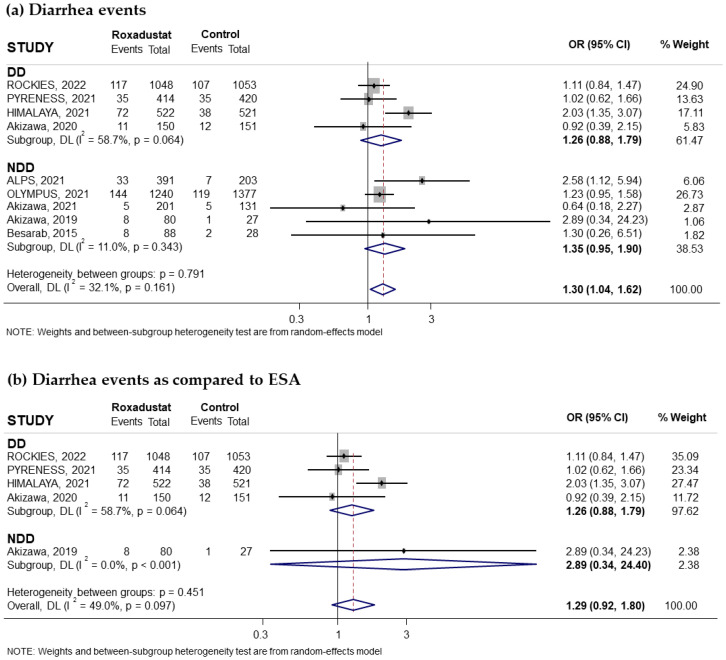

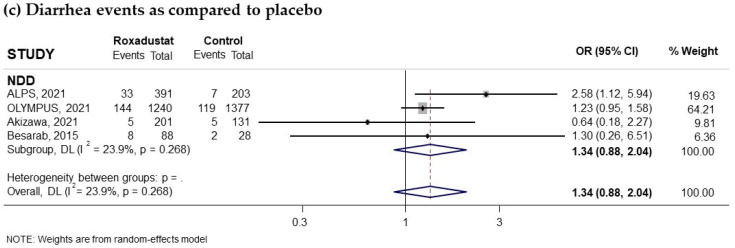
Incidence of diarrhea events by type of comparator [14,18,23,24,26,27,28,29,31]. Forest plot of the effect of Roxadustat on diarrhea events in anemic patients with CKD depending on whether they are on dialysis or not, according to the type of comparator. (**a**) Patients with diarrhea events by study population, dialysis-dependent (DD) or non-dialysis-dependent (NDD) patients, comparing Roxadustat with the control group. Patients with diarrhea events analyzed by subgroups according to comparator, ESA (**b**) or placebo (**c**), within each population (DD or NDD). All results are presented as odds ratios (ORs) for treatment versus comparator, with their 95% confidence intervals (95% CIs).

**Figure 11:**
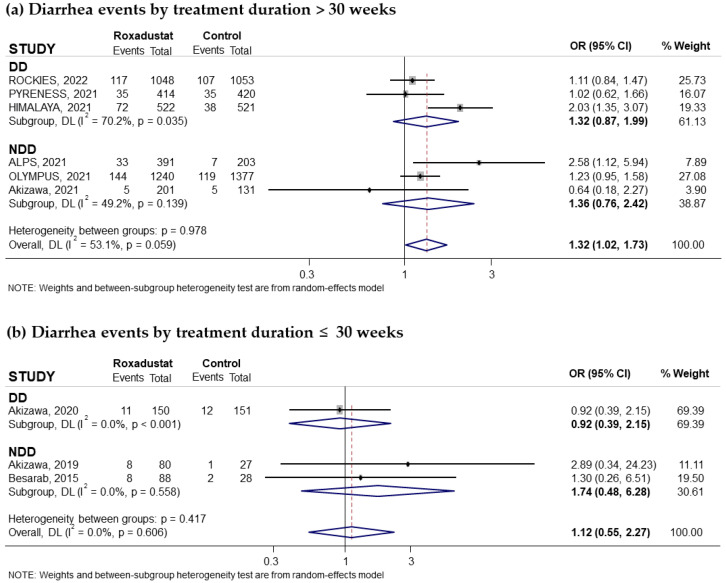
Incidence of diarrhea events by duration of treatment with Roxadustat [14,18,23,24,26,27,28,29,31]. Forest plot of the effect of Roxadustat on diarrhea events in anemic patients with CKD depending on whether they are on dialysis or not, according to the duration of treatment with Roxadustat. Patients with diarrhea events analyzed according to the duration of treatment with Roxadustat, >30 weeks (**a**) or ≤30 weeks (**b**), within each population, dialysis-dependent (DD) or non-dialysis-dependent (NDD) patients. All results are presented as odds ratios (ORs) for treatment versus comparator, with their 95% confidence intervals (95% CIs).

Nine trials reporting the risk of diarrhea associated with oral Roxadustat treatment for anemia in CKD patients were included in our study. Five of these trials had recruited non-dialysis-dependent patients and four had recruited dialysis patients. Overall, a significantly increased risk was observed with the use of Roxadustat, with a pooled OR of 1.30 (95% CI: 1.04–1.62; I^2^ = 32.1%) (Figure 10a). However, subgroup analysis according to whether patients were on dialysis or not did not reflect significant changes in the risk of diarrhea (Figure 10a). Subgroup analysis based on the comparator used did not reflect an increased risk of diarrhea compared with ESAs (Figure 10b) or placebo (Figure 10c). According to duration of treatment, we found a significant increased risk of diarrhea if the treatment duration was longer than 30 weeks (pooled OR = 1.32; 95% CI: 1.02–1.73) with moderate heterogeneity (I^2^ = 53.1%) (Figure 11a) but not when the treatment duration was less than 30 weeks (pooled OR = 1.12; 95% CI: 0.55–2.27; I^2^ = 0.0%) (Figure 11b). However, when assessing the heterogeneity of long-term treatment, no significant increased risk was observed in any of the subgroups based on whether or not they received dialysis (Figure 11a). No evidence of publication bias was observed, as assessed by funnel plot (Figure S1e). A Begg and Mazumdar correlation test was not performed, as there were only nine studies.

#### 3.3.4. Effect of Roxadustat on Hyperkalemia

**Figure 12:**
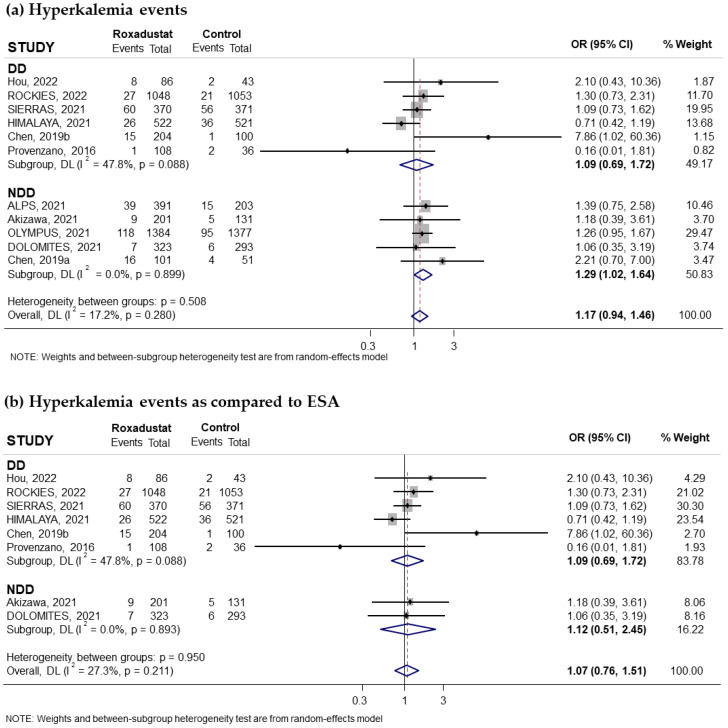

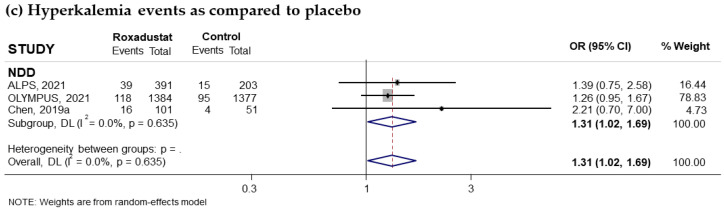
Incidence of hyperkalemia events by type of comparator [16,17,22,24,25,26,27,28,30,31,32]. Forest plot of the effect of Roxadustat on hyperkalemia events in anemic patients with CKD depending on whether they are on dialysis or not, according to the type of comparator. (**a**) Patients with hyperkalemia events by study population, dialysis-dependent (DD) or non-dialysis-dependent (NDD) patients, comparing Roxadustat with the control group. Patients with hyperkalemia events analyzed by subgroups according to comparator, ESA (**b**) or placebo (**c**), within each population (DD or NDD). All results are presented as odds ratios (ORs) for treatment versus comparator, with their 95% confidence intervals (95% CIs).

**Figure 13:**
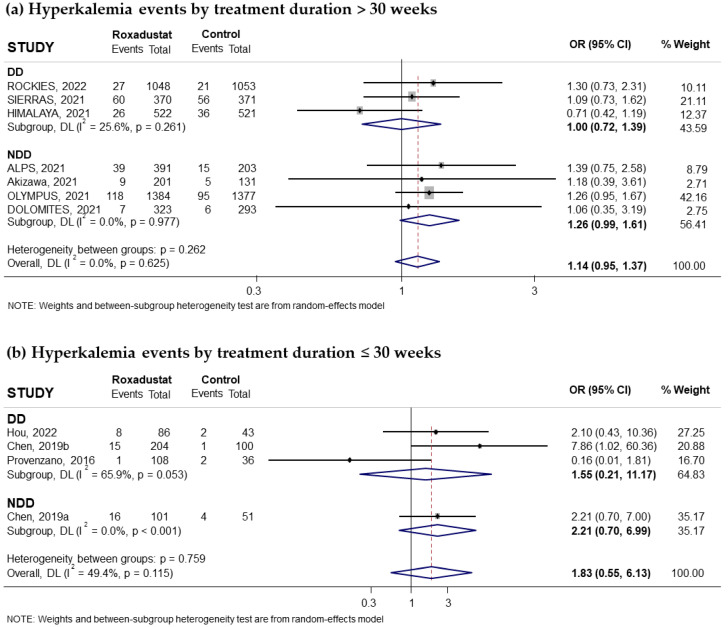

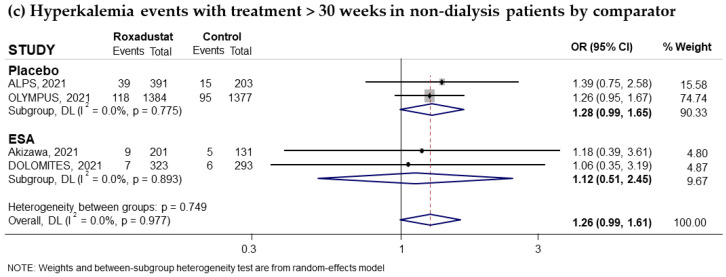
Incidence of hyperkalemia events by duration of treatment with Roxadustat [16,17,22,24,25,26,27,28,30,31,32]. Forest plot of the effect of Roxadustat on hyperkalemia events in anemic patients with CKD depending on whether they are on dialysis or not, according to the duration of treatment with Roxadustat. Patients with hyperkalemia events analyzed according to the duration of treatment with Roxadustat, >30 weeks (**a**) or ≤30 weeks (**b**), within each population, dialysis-dependent (DD) or non-dialysis-dependent (NDD) patients. (**c**) Patients with hyperkalemia events analyzed in NDD patients with treatment > 30 weeks according to the type of comparator, ESA or placebo. All results are presented as odds ratios (ORs) for treatment versus comparator, with their 95% confidence intervals (95% CIs).

Eleven trials reporting the risk of hyperkalemia associated with oral Roxadustat treatment for anemia in CKD patients were included in our study. Five of these trials had recruited non-dialysis-dependent patients and six had recruited dialysis patients. The pooled OR was 1.17 (95% CI: 0.94–1.46; I^2^ = 17.2%) (Figure 12a). Subgroup analysis based on whether patients were on dialysis or not suggested that the increased risk of hyperkalemia was statistically significant in non-dialysis-dependent patients (pooled OR = 1.29; 95% CI: 1.02–1.64; I^2^ = 0.0%) (Figure 12a), while it was not statistically significant in the group of patients on dialysis (pooled OR = 1.09; 95% CI: 0.69–1.72) with moderate heterogeneity (I^2^ = 47.8%) (Figure 12a). Subgroup analysis according to the comparator used did not show a statistically significant increase in risk compared to ESAs (pooled OR = 1.07; 95% CI: 0.76–1.51; I^2^ = 27.3%) (Figure 12b) but did show an increased risk compared to placebo (pooled OR = 1.31; 95% CI: 1.02–1.69; I^2^ = 0.0%) (Figure 12c). Similarly, when we analyzed the data according to the duration of Roxadustat treatment, an increased risk was observed with both longer and shorter treatments than 30 weeks, but this increase did not reach statistical significance in either case (Figure 13a,b). Notably, the subgroup of patients without dialysis and with treatment > 30 weeks had a near-significant increased risk of hyperkalemia compared to placebo (pooled OR = 1.28; 95% CI: 0.99–1.65; I^2^ = 0.0%) but not compared to ESAs (Figure 13c). No evidence of publication bias was observed, as assessed by the funnel plot and Begg and Mazumdar correlation, *p* = 0.311 (Figure S1f).

### 3.4. Analysis of the Impact of Risk of Bias on the Results

Following risk-of-bias assessment, most studies were classified as ‘low risk’ for randomization and outcome measurement. However, some studies showed ‘some concerns’ or ‘high risk’ of deviations from the intended interventions and missing outcome data, especially in open-label trials (eight of the fifteen included studies, representing 40% of the patients) or those with unequal follow-up between the experimental and control groups (Table S1).

A sensitivity analysis excluding the open-label trials was performed, and the results remained generally quite consistent with those found in this meta-analysis for each adverse event (MACE, hypertension, diarrhea, and hyperkalemia). The results of the sensitivity analysis are presented in the Supplementary Material (Figure S4).

## 4. Discussion

In this study, a systematic review and meta-analysis of clinical trials reporting information on the safety of Roxadustat were conducted to assess the impact of the most common adverse events affecting anemic patients with CKD, both on and off dialysis, and considering the duration of treatment. Meta-analysis of the 15 trials included in this study revealed no significant differences in events related to MACEs between the Roxadustat group and either the placebo or ESA group.

In general, Roxadustat was associated with a higher incidence of adverse events in non-dialysis patients, with a significant increase in SAEs, hypertension, and hyperkalemia, and a borderline increase in AEs and diarrhea. The incidence of adverse events also increased when anemic patients with chronic kidney disease received Roxadustat for more than 30 weeks, especially SAEs, hypertension, and diarrhea. The study by Tang et al. also observed a higher incidence of SAEs in the group of patients not undergoing dialysis [33]. Regarding the most common adverse events, a higher risk of hyperkalemia or elevated potassium levels was observed in the oral Roxadustat treatment group in non-dialysis patients compared to the placebo group, noting that this risk tends to continue if treatment is prolonged for more than 30 weeks. Similarly, an increased risk of hypertension was observed in patients taking oral Roxadustat but not on dialysis, especially during long-term treatment. The risk of hyperkalemia or hypertension was not evident in the group of patients who took oral Roxadustat and received dialysis, even during long-term treatment. In agreement with these results, several meta-analyses show similar findings regarding an increased incidence of hypertension, hyperkalemia, or diarrhea in anemic patients with CKD treated with Roxadustat, although all confirm its efficacy in the treatment of anemia [34,35,36,37,38,39]. Overall, they reveal that Roxadustat does not produce significant differences in cardiovascular events compared with the control group but increases the risk of hypertension [34,35,36,37], hyperkalemia [34,35,36], and diarrhea [36]. These changes were more evident in the placebo group than in the ESA group. In contrast, other meta-analyses show that Roxadustat does not present a higher risk of adverse events than other ESAs [40,41], and is even safer with regard to MACEs [42]. Bartnicki et al. [43] also found that the most common adverse effects associated with HIF-PHs inhibitors treatment were diarrhea, hyperkalemia, and hypertension, among others [35].

This meta-analysis also identified an increased risk of diarrhea in patients receiving oral Roxadustat compared to the control group. The risk of diarrhea increased with longer treatment periods of more than 30 weeks.

Currently, there is no comprehensive research on the effects of Roxadustat on blood potassium levels. The effect of Roxadustat on blood potassium levels may vary between individuals. Roxadustat is primarily metabolized in the liver and excreted primarily in the bile, while a small portion is eliminated by the kidneys [34,44]. Patients with CKD often have elevated blood potassium levels because potassium accumulates when the kidneys do not properly eliminate it. This hyperkalemia can be life-threatening by increasing arrhythmias if potassium levels are not controlled. In certain cases, Roxadustat may also interfere with renal potassium excretion, which could result in elevated blood potassium levels and increased risk to the patient. However, no reliable evidence is currently available. Concerns have been raised that hyperkalemia (serum potassium concentrations > 5.5 mmol/L) may be associated with the use of HIF-PHs inhibitors. However, while some authors describe a higher incidence of hyperkalemia in NDD-CKD patients treated with roxadustat compared with placebo [16,26,28], or in DD-CKD patients treated with roxadustat compared with ESAs [45], others, conversely, find a higher incidence of hyperkalemia in patients treated with ESAs compared with roxadustat [18,25,27]. Vadadustat also did not show a higher incidence of hyperkalemia compared with ESAs [46,47]. Although the literature data on the incidence of hyperkalemia in patients treated with HIF-PHs inhibitors are inconclusive, the possibility that hyperkalemia may be life-threatening cannot be ruled out; therefore, monitoring of blood potassium levels before and during HIF-PHs inhibitors treatment is recommended [48]. Hyperkalemia associated with Roxadustat treatment is thought to be related to metabolic acidosis [13], a common condition in patients with chronic kidney disease, in which potassium loss from the intracellular to the extracellular space is increased. In diarrhea, gastrointestinal losses of bicarbonate and potassium are common. However, if diarrhea is associated with a greater deterioration in renal function than that already present in patients with chronic kidney disease, the risk of hyperkalemia may be increased, since renal potassium losses are limited. In this regard, studies by Chang et al. show that Roxadustat, in addition to inhibiting HIF-PHs, is itself capable of interacting directly with membrane ion channels, thereby altering the amplitude and activation of ionic currents. It potently and effectively suppresses ionic currents through a non-genomic pathway, but being an acute effect, it is probably not related to HIF-1α activity [49]. When cells were exposed to cobalt chloride or deferoxamine, the ionic current induced by membrane depolarization was unchanged; however, nonactin, a K^+^-selective ionophore, in the continuous presence of roxadustat, attenuated the inhibition of roxadustat-mediated amplitude.

Regarding the effect of Roxadustat on hypertension, our results indicated an increased risk of hypertension in non-dialysis patients. It is known that hypoxia-inducible factor may contribute to the development of pulmonary hypertension, since HIF regulates genes encoding peptides such as endothelin-1, a potent vasoconstrictor peptide, but it also regulates genes such as iNOS that produces nitric oxide, a potent vasodilatory peptide. This suggests that PHs inhibitors such as Roxadustat have a complex effect on the vasculature [34]. Yuan et al. [50] studied the possible therapeutic effects derived from both activation and inhibition of HIF, and reports that HIF inhibition could serve as a therapy in pulmonary hypertension [50]. Consistent with our study, Zhang et al. [51] also observed that HIF-PHs inhibitors were associated with a higher risk of hypertension than placebo, especially in CKD patients not on dialysis, but this risk was lower than with ESA treatment. The mechanisms of the increased risk of hypertension associated with HIF-PHs inhibitors use remain under investigation. It is well known that treatment with EPO increases blood pressure and the risk of hypertension [52], but the mechanism is unclear. Compared with EPOs, HIF stabilizers might be less pressor-like than EPO alone, as several genes in the HIF-mediated transcriptional cascade are involved in vasomotor control [53], although they also upregulate EPO genes. In animal studies, hypoxia induced vascular smooth muscle calcification, which might contribute to arterial stiffness and subsequently hypertension. Furthermore, chronic intermittent hypoxia through HIF signaling is thought to lead to systemic hypertension [54]. However, animal studies with roxadustat did not affect the development of hypertension or cardiac hypertrophy in salt-sensitive Dahl rats on a high-salt diet; however, it attenuated renal fibrosis in these rats [55]. In contrast, previous studies showed that treatment with roxadustat markedly improved hypertension and organ injury in an angiotensin II model of hypertension, possibly through stabilization of HIF1α and subsequent targeting of endothelial nitric oxide synthase (eNOS), AGTR1, AGTR2, and oxidative stress [56]. Recent studies show a possible beneficial effect of Roxadustat at the cardiovascular level by inducing vasorelaxation [57]. These in vivo studies in rats showed that roxadustat not only relaxed the thoracic aorta, but also the superior mesenteric artery, a smaller vessel. The vasorelaxant effects of roxadustat in the thoracic aorta were likely mediated by the activation of eNOS through bradykinin B2 receptors and the subsequent activation of the NO/sGC pathway, as well as by the opening of potassium channels expressed in smooth muscle, such as the ATP-sensitive potassium channel (KATP), the high-conductance calcium-activated potassium channel (BKCa), and the voltage-gated potassium channel (Kv), which cause vasorelaxation by hyperpolarization [58]. Conversely, the closure of potassium channels induces vasocontraction by depolarization. They suggest that administration of roxadustat to patients with CKD could have beneficial effects not only on renal anemia but also on the vasculature. In this regard, chronic administration of roxadustat to a mouse model of angiotensin II-induced hypertension has been shown to suppress hypertension and kidney damage through eNOS activation associated with HIF stabilization [56] and even showed a slight reduction in blood pressure and cardiovascular protection [59,60]. Regarding the relationship between HIF and eNOS, eNOS expression is increased by exposure to hypoxia through HIF-2 [61] and by chronic administration of roxadustat through HIF-1α [56]; however, since roxadustat-induced vasorelaxation is an acute response, the authors believe that it is unlikely that the effect is mediated by the regulation of HIF gene expression. This potential vasorelaxant effect of Roxadustat reported in vivo goes against our results, where after analyzing 15 clinical trials, long-term Roxadustat is observed to increase the risk of hypertension. Therefore, current research on the effects of Roxadustat on blood pressure and its possible mechanisms need further studies with larger sample sizes to carry out in-depth and systematic investigations in the future.

Long-term use of PHs inhibitors could have adverse effects due to the persistent activation of HIF-regulated genes. Roxadustat is a first-in-class HIF-PH inhibitor that works by stabilizing HIF, preventing its degradation by blocking HIF-alpha hydroxylation, thereby promoting the erythropoietic response. However, potential risks related to angiogenesis, carcinogenesis, and tumorigenesis associated with HIF stabilization have also been hypothesized. Specifically, vascular endothelial growth factor activation is involved in various biological processes, such as tumor growth [62], cell differentiation, and mitochondrial metabolism [63]. The adverse effects of roxadustat might be explained at least in part by the fact that this hypoxia mimetic can potentiate both HIF-1α and HIF-2α, although it preferentially acts on HIF-2α [64]. Hypoxia mimetics potentiate HIF-1α and/or HIF-2α activity by inhibiting one of the three PHs responsible for the degradation of the HIF isoforms. Inhibition of PH2 primarily potentiates HIF-1α activity [65], whereas PH3 preferentially potentiates HIF-2α [66,67,68]. Cobalt chloride and roxadustat are hypoxia mimetics that increase erythropoietin production and can treat anemia by inhibiting PH3 [65,69,70], although they can also suppress other PHs (depending on the dose). HIF-1α and HIF-2α exert mutually antagonistic effects on redox status and proinflammatory pathways. The short-term actions of HIF-1α reduce hypoxic injury, but the long-term effects cause increased oxidative stress, inflammation, and fibrosis; however, these deleterious effects are counteracted by the actions of HIF-2α. However, this potential mechanism has not been demonstrated in the populations studied, and additional studies with long-term follow-up would be required before definitive conclusions can be drawn.

The findings of this meta-analysis on the safety of roxadustat highlight the importance of caution and vigilance when treating these patients, including monitoring blood pressure and serum potassium levels, to prevent the occurrence of these common adverse effects.

### Limitations and Strengths

This meta-analysis has several limitations. Different doses and durations of Roxadustat may affect HIF regulation in multiple target genes to varying degrees, which could affect the results of the analysis. Mortality caused by Roxadustat in older patients cannot be estimated from the data of the published studies. Although all studies included subjects between 18 and 75–80 years, the proportion of patients over 70 years was small in all trials. Therefore, the results of this study may not be transported to the very old patients.

After performing the risk-of-bias assessment, several studies were rated as having “some concerns” or “high risk.” This was largely due to the open-label design, which may have led to differential reporting of subjective adverse events between the experimental and control groups, potentially exaggerating adverse events in the experimental arm. Additionally, the duration follow-up was often shorter in the control group (28 to 59 weeks) compared with the experimental group (46 to 70 weeks), which could result in underreporting of adverse events in the control arm. Although I^2^ was low in most analyses, heterogeneity in some outcomes, related to differences in study design, could affect the validity of the pooled results. These limitations should be considered when interpreting the results; however, the sensitivity analysis excluding open-label trials yielded consistent findings, suggesting that the overall conclusions of this meta-analysis are robust.

Despite these limitations, this systematic review and meta-analysis is novel, as most studies prioritized the efficacy of Roxadustat compared with erythropoiesis-stimulating agents or placebo, and safety outcomes were secondary. All reported that Roxadustat had equal or superior efficacy to ESAs. However, regarding the safety profile of Roxadustat, the authors simply concluded that the observed data were consistent with previous studies conducted in a CKD patient population, but few emphasized the most common events that jeopardize patient safety.

This meta-analysis revealed that, overall, Roxadustat increased the incidence of serious adverse events, with no significant differences detected in MACEs. In contrast, an increased risk of common events such as hypertension and hyperkalemia was identified in non-dialysis patients compared with the placebo group. In long-term treatment, the risk of SAEs, hypertension, and diarrhea was notable, while the risk of hyperkalemia remained borderline significant.

## 5. Conclusions

Roxadustat is a new treatment for anemia in CKD, with efficacy equal to or superior than ESAs. However, data on its safety are limited, and the present meta-analysis, which includes 15 randomized controlled trials and a total of 10,284 patients, seeks to contribute to the understanding of this important topic. The study shows an increased risk of adverse events, including SAEs, hypertension, and hyperkalemia, especially in CKD patients not receiving dialysis, compared to placebo. Importantly, no increased risk of MACEs was observed. No differences in the incidence of SAEs and AEs were observed compared to ESAs, the currently recommended therapy for the treatment of anemia in these patients. Prolonged exposure to Roxadustat treatment, exceeding 30 weeks, is correlated with an increased risk of adverse effects, such as serious adverse events, hypertension, and diarrhea. However, further long-term studies are required to confirm this time-dependent toxicity profile, which is not observed in short-term trials, given the chronic nature of anemia treatments in CKD patients. Caution is recommended when treating anemia in patients with chronic kidney disease not yet receiving dialysis and with a long-term treatment, by monitoring blood pressure, blood potassium levels, and the occurrence of any adverse events to reduce the dose of Roxadustat or, if necessary, discontinue treatment.

## Figures and Tables

**Table 1:** Subject demographics and baseline characteristics of the studies included in the meta-analysis.

Scheme	1.	Phase of Study, Location	Number of Patients	Gender (%)	Age Range (Years)	Intervention: ROXADUSTAT (Dose)	Control	Duration of Study (Weeks)	Patient Disease	Baseline Hb ^1^ (g/dL)
Besarab et al., 2015 [14]	NCT00761657	II, USA	116	Male (42) Female (58)	18−80 Mean: 65.8	0.7, 1, 1.5 or 2 mg/kg	Placebo	4	Anemia in patients with stages 3–4 CKD who were not receiving dialysis.	10.3 ± 0.9 10.3 ± 0.9
Chen et al., 2019 [16]	NCT02652819	III, China	152	Male (37) Female (63)	18–75 Mean: 54	70 or 100 mg	Placebo	9	Anemia in patients with stages 3–5 CKD who were not receiving dialysis.	8.9 ± 0.8 8.9 ± 0.7
Akizawa et al., 2019 [23]	NCT01964196	II, Japan	107	Male (47) Female (53)	20–74 Mean: 63.15	50, 70 or 100 mg	Placebo	24	Anemia and CKD who were not receiving dialysis.	9.4 ± 0.6 9.3 ± 0.7
Akizawa et al., 2021 [24]	NCT02988973	III, Japan	334	Male (58) Female (42)	≥20 Mean: 70 <65 años (26%) >65 años (74%)	Initial dose conversion to roxadustat, based on ESA use	Darbopoetin alfa	52	Anemia and CKD who were not receiving dialysis	11.03 ± 0.56 10.96 ± 0.52
Shutov et al., 2021 [26]	NCT01887600 ALPS	III, Russia	594	Male (45) Female (55)	≥18 Mean: 62.5	70 or 100 mg	Placebo	52	Anemia in patients with stages 3–5 CKD, who were not receiving dialysis.	9.1 ± 0.8 9.1 ± 0.7
Fishbane et al., 2021 [28]	NCT02174627 OLYMPUS	III, USA	2761	Male (42) Female (58)	≥18 Mean: 61.7	70 mg	Placebo	52 weeks up to 4 years	Anemia in patients with stages 3–5 CKD who were not receiving dialysis.	9.1 ± 0.7 9.1 ± 0.7
Barratt et al., 2021 [25]	NCT02021318 DOLOMITES	III, Europe	616	Male (44) Female (56)	≥18 Mean: 66.3	70 or 100 mg	Darbopoetin alfa	104	Anemia in patients with stages 3–5 CKD who were not receiving dialysis.	9.55 ± 0.75 9.55 ± 0.69
Provenzano et al., 2016 [22]	NCT01147666	II, USA	144	Male (66) Female (34)	18–75 Mean: 57.3	1, 1.5, 1.8 or 2 mg/kg	Epoetin alfa	6–19	Anemia in patients with end-stage CKD on stable HD.	11.2 ± 0.7 11.2 ± 1.0
Chen et al., 2019 [17]	NCT02652806	III, China	304	Male (61) Female (39)	18–75 Mean: 49.3	100 or 120 mg	Epoetin alfa	27	Anemia in patients with stages 3–5 CKD on stable HD (89%) or PD (11%).	10.4 ± 0.7 10.5 ± 0.7
Akizawa et al., 2020 [18]	NCT02952092	III, Japan	303	Male (67) Female (33)	≥20 Mean: 64.75	70 or 100 mg	Darbopoetin alfa	24	Anemia and CKD on stable HD.	11.0 ± 0.6 11.0 ± 0.6
Provenzano et al., 2021 [27]	NCT02052310 HIMALAYAS	III, USA, Asia, South America, Europe	1043	Male (59) Female (41)	≥18 Mean: 54	70 or 100 mg	Epoetin alfa	89	Anemia and CKD on stable HD (89%) or PD (11%).	8.4 ± 1.0 8.5 ± 1.0
Charytan et al., 2021 [30]	NCT02273726 SIERRAS	III, USA	741	Male (54) Female (46)	≥18 Mean: 58	70, 100, 150 or 200 mg	Epoetin alfa	28–52	Anemia and CKD on stable HD (96%) and PD (4%)	10.30 ± 0.66 10.31 ± 0.66
Csiky et al., 2021 [29]	NCT02278341 EudraCT2013-001497-16 PYRENESS	III, Europe	834	Male (58) Female (42)	≥18 Mean: 61.4	20, 50 or 100 mg	Epoetin alfa or Darbopoetin alfa	52–104	Anemia in patients with end-stage CKD on stable HD (94%) or PD (6%).	10.75 ± 0.62 10.78 ± 0.62
Hou et al., 2022 [32]	ChiCTR2000035054	China	129	Male (56) Female (44)	18–75 Mean: 48.2	100 or 120 mg	ESA	24	Anemia and CKD on stable PD.	9.0 ± 1.4 9.0 ± 1.2
Fishbane et al., 2022 [31]	NCT02174731 ROCKIES	III, USA, Asia, Latin America, Europe and Australia,	2106	Male (59) Female (41)	≥18 Mean: 54	70, 100 or 200 mg	Epoetin alfa	Up to 4 years	Anemia in patients with end-stage CKD on stable HD (89%) or PD (11%).	10.2 (9.3–10.9) 10.3 (9.2–11.0)

^1^ Baseline of hemoglobin (Hb) is expressed as the mean ± SD in all studies except the last one, which is expressed as the median (IQR).

## Data Availability

The protocol for this meta-analysis has not been registered; this is a systematic review of several previously registered clinical trials published in the literature [14,16,17,18,20,21,22,23,24,25,26,27,28,29,30]. Data presented in this study is contained within the article and supplementary material.

## Supplementary Materials

The following supporting information can be downloaded at https://www.mdpi.com/article/10.3390/ph18101566/s1, Figure S1: Funnel plots to assess publication bias; Figure S2: Forest plot of the effect of Roxadustat treatment duration on the incidence of SAEs in anemic patients with CKD, according to the comparator; Figure S3: Forest plot of the effect of Roxadustat for more than 30 weeks on the incidence of MACEs in anemic patients with CKD, according to the comparator; Figure S4: Forest plot of the effect of Roxadustat on the incidence of adverse effects in anemic patients with CKD, depending on whether they are on dialysis or not, after excluding open-label clinical trials; and Table S1: Risk of bias in included RCTs.

## Abbreviations

The following abbreviations are used in this manuscript:

AEs	Adverse Events
CIs	Confidence Intervals
CKD	Chronic Kidney Disease
DD	Dialysis-dependent
EPO	Erythropoietin
ESAs	Erythropoietic-Stimulating Agents
Hb	Hemoglobin
HD	Hemodialysis
HIF	Hypoxia-Inducible Factor
MACEs	Major Adverse Cardiovascular Events
NDD	Non-dialysis-dependent
OR	Odds Ratio
PD	Peritoneal Dialysis
PHs	Prolyl hydroxylases
SAEs	Serious Adverse Events
