# Supplementary material for: Safety of Roxadustat in Chronic Kidney Disease Patients: An Updated Systematic Review and Meta-Analysis
Source: Pharmaceuticals (Basel). 2025 Oct 17;18(10):1566. doi: 10.3390/ph18101566 (PMC12567109; doi:10.3390/ph18101566)
Supplement: Supplementary file 1 [file pharmaceuticals-18-01566-s001.zip › Supplemental material-Figure S3.pdf]

**(a) MACE events by treatment duration >30 weeks in NDD patients**

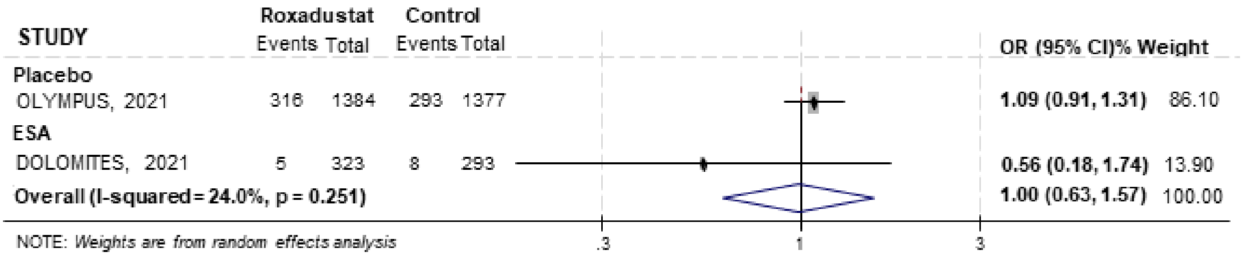


**(b) MACE events by treatment duration >30 weeks in DD patients**

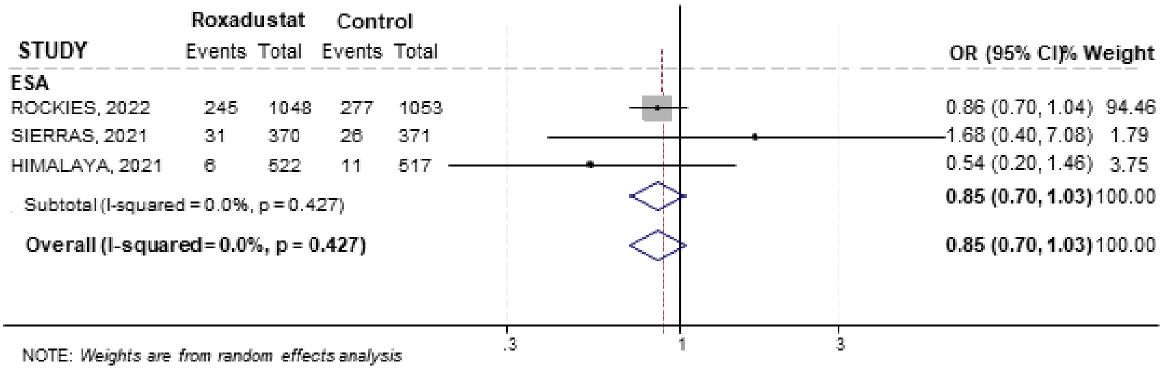


**Figure S3. Forest plot of the effect of Roxadustat for more than 30 weeks on the incidence of MACE in anemic patients with CKD, according to the comparator. (a)** Patients with MACE events analyzed in patients not receiving dialysis with treatment > 30 weeks according to the type of comparator, ESA or placebo. **(b)** Patients with MACE events analyzed in patients on dialysis with treatment > 30 weeks according to the comparator. All results are presented as odds ratios (ORs) for treatment versus comparator, with their 95% confidence intervals (95% CI).
