# Supplementary material for: Safety of Roxadustat in Chronic Kidney Disease Patients: An Updated Systematic Review and Meta-Analysis
Source: Pharmaceuticals (Basel). 2025 Oct 17;18(10):1566. doi: 10.3390/ph18101566 (PMC12567109; doi:10.3390/ph18101566)
Supplement: Supplementary file 1 [file pharmaceuticals-18-01566-s001.zip › Supplemental material-Figure S4.pdf]

(a) Incidence of MACE events by type of comparator excluding open-label clinical trials

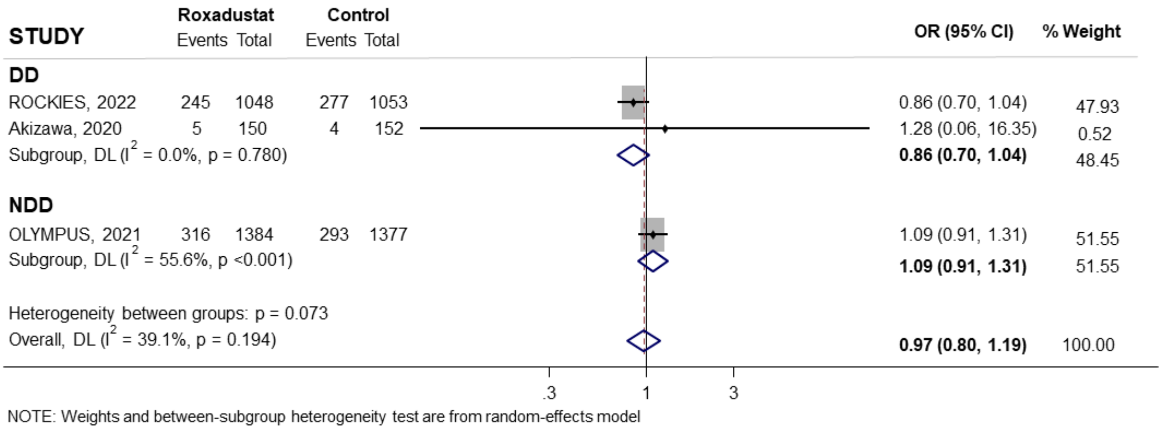

(b) Incidence of hypertension events by type of comparator excluding open-label clinical trials

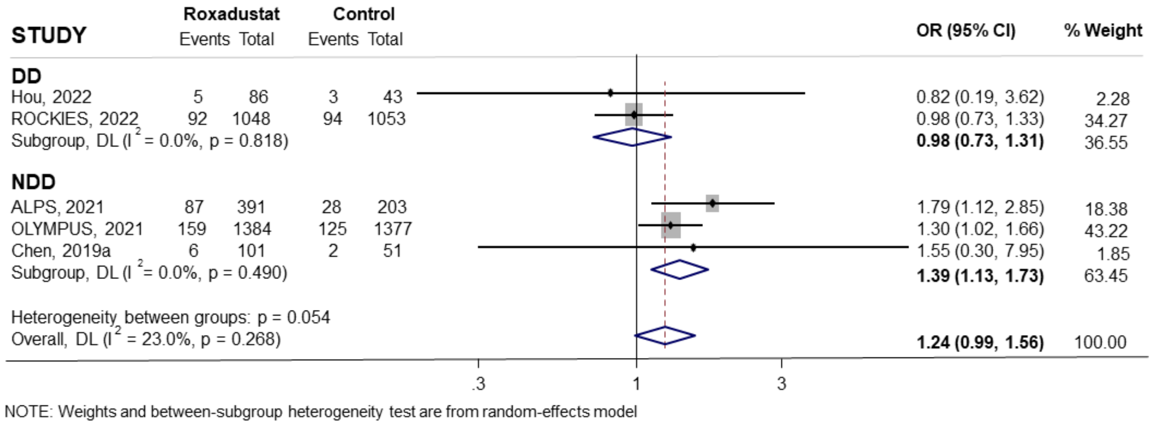

(c) Incidence of diarrhea events by type of comparator excluding open-label clinical trials

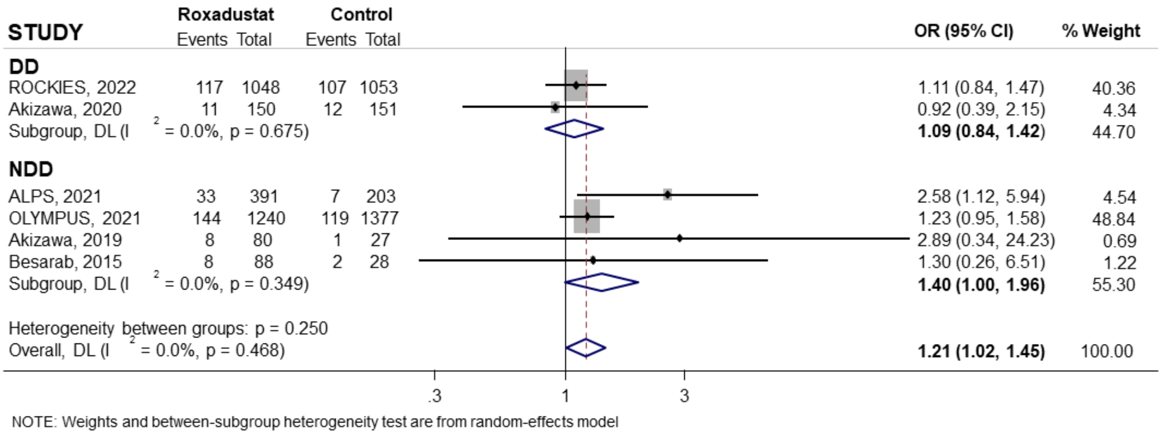

(d) Incidence of hyperkalemia events by type of comparator excluding open-label clinical trials

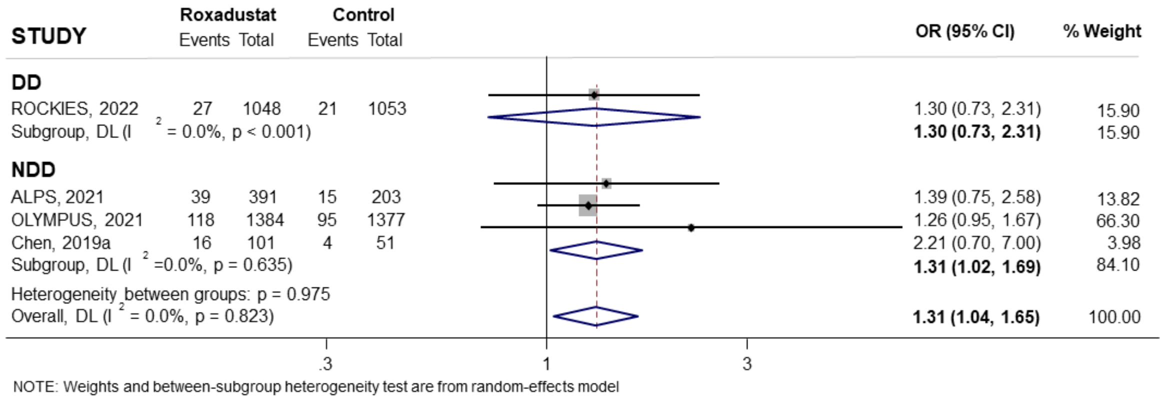

Figure S4. Forest plot of the effect of Roxadustat on the incidence of adverse events in anemic patients with CKD, depending on whether they are on dialysis or not, after analysis excluding open-label clinical trials. (**a**) Patients with MACE events, (**b**) patients with hypertension events, (**c**) patients with diahrrea events, and (**d**) patients with hyperkalemia events. All results are presented as odds ratios (ORs) for treatment versus comparator, with their 95% confidence intervals (95% CI).
