# Supplementary material for: Safety of Roxadustat in Chronic Kidney Disease Patients: An Updated Systematic Review and Meta-Analysis
Source: Pharmaceuticals (Basel). 2025 Oct 17;18(10):1566. doi: 10.3390/ph18101566 (PMC12567109; doi:10.3390/ph18101566)
Supplement: Supplementary file 1 [file pharmaceuticals-18-01566-s001.zip › Supplemental material-Table S1.pdf]

Table S1. Risk of Bias (Cochrane RoB 2.0) in included RCTs.

Study	Clinical Trial number	Randomization process	Deviations from intended interventions	Missing outcome data	Outcome measurement	Selection of the reported result
Besarab et al, 2015 [14]	NCT00761657	Some concerns	Some concerns	Low risk	Some concerns	Low risk
Chen et al, 2019[a,] [16]	NCT02652819	Low risk	Low risk	Some concerns	Low risk	Low risk
Akizawa et al, 2019 [21]	NCT01964196	Low risk	Low risk	Some concerns	Low risk	Low risk
Akizawa et al, 2021 [22]	NCT02988973	Some concerns	High risk	Some concerns	Low risk	Low risk
Shutov et al, 2021 [24]	NCT01887600 ALPS	Low risk	Low risk	Some concerns	Low risk	Low risk
Fishbane et al, 2021 [26]	NCT02174627 OLYMPUS	Low risk	Low risk	Some concerns	Low risk	Low risk
Barratt et al, 2021 [23]	NCT02021318 DOLOMITES	Some concerns	High risk	Low risk	Some concerns	Low risk

Table S1 (continued)

Study	Clinical Trial number	Randomization process	Deviations from intended interventions	Missing outcome data	Outcome measurement	Selection of the reported result
Provenzano et al, 2016 [20]	NCT01147666	Some concerns	High risk	Some concerns	Some concerns	Some concerns
Chen et al, 2019[b] [17]	NCT02652806	Some concerns	High risk	Some concerns	Some concerns	Some concerns
Akizawa et al, 2020 [18]	NCT02952092	Low risk	Low risk	Low risk	Low risk	Low risk
Provenzano et al, 2021 [25]	NCT02052310 HIMALAYAS	Low risk	High risk	Some concerns	Some concerns	Some concerns
Charytan et al, 2021 [28]	NCT02273726 SIERRAS	Some concerns	High risk	Some concerns	Some concerns	Some concerns
Csiky et al, 2021 [27]	NCT02278341 EudraCT2013-001497-16 PYRENESS	Some concerns	High risk	Some concerns	Low risk	Some concerns
Hou et al, 2022 [30]	ChiCTR2000035054	Some concerns	High risk	Low risk	Some concerns	Some concerns
Fishbane et al, 2022 [29]	NCT02174731 ROCKIES	Low risk	Some concerns	Some concerns	Low risk	Low risk
