# Supplementary material for: Safety of Roxadustat in Chronic Kidney Disease Patients: An Updated Systematic Review and Meta-Analysis
Source: Pharmaceuticals (Basel). 2025 Oct 17;18(10):1566. doi: 10.3390/ph18101566 (PMC12567109; doi:10.3390/ph18101566)
Supplement: Supplementary file 1 [file pharmaceuticals-18-01566-s001.zip › Supplemental material-Figure S1.pdf]

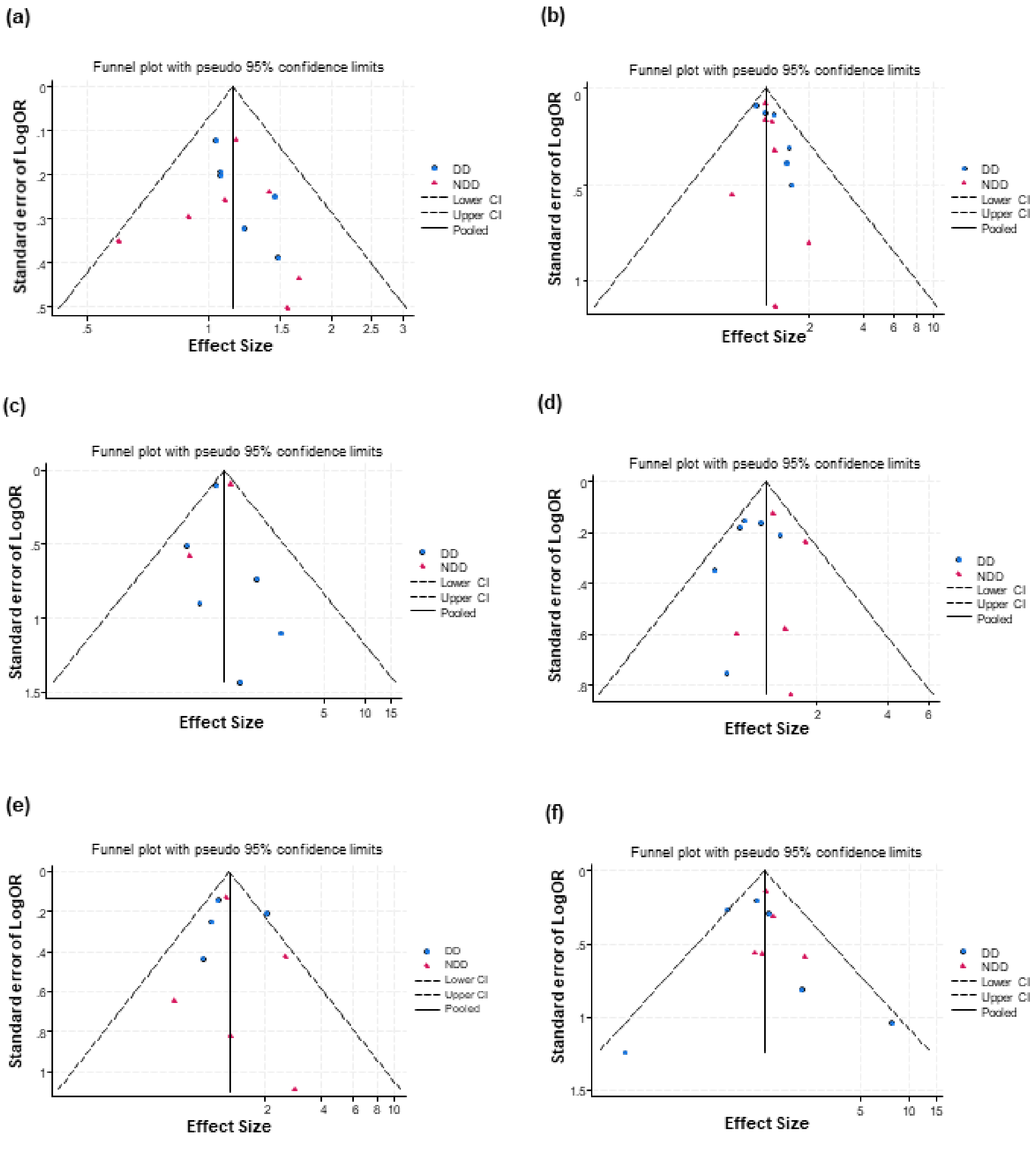

Figure S1. Funnel plots to assess publication bias. (a) Adverse events, **(b)** serious adverse events, **(c)** MACE, **(d)** hypertension, **(e)** diarrhea, and **(f)** hyperkalemia. Publication bias was calculated using the Begg and Mazumdar adjusted rank correlation method; it was excluded if $P > 0.05$.
