# Supplementary material for: Safety of Roxadustat in Chronic Kidney Disease Patients: An Updated Systematic Review and Meta-Analysis
Source: Pharmaceuticals (Basel). 2025 Oct 17;18(10):1566. doi: 10.3390/ph18101566 (PMC12567109; doi:10.3390/ph18101566)
Supplement: Supplementary file 1 [file pharmaceuticals-18-01566-s001.zip › Supplemental material-Figure S2.pdf]

(a) Serious adverse events by treatment duration > 30 weeks in NDD patients

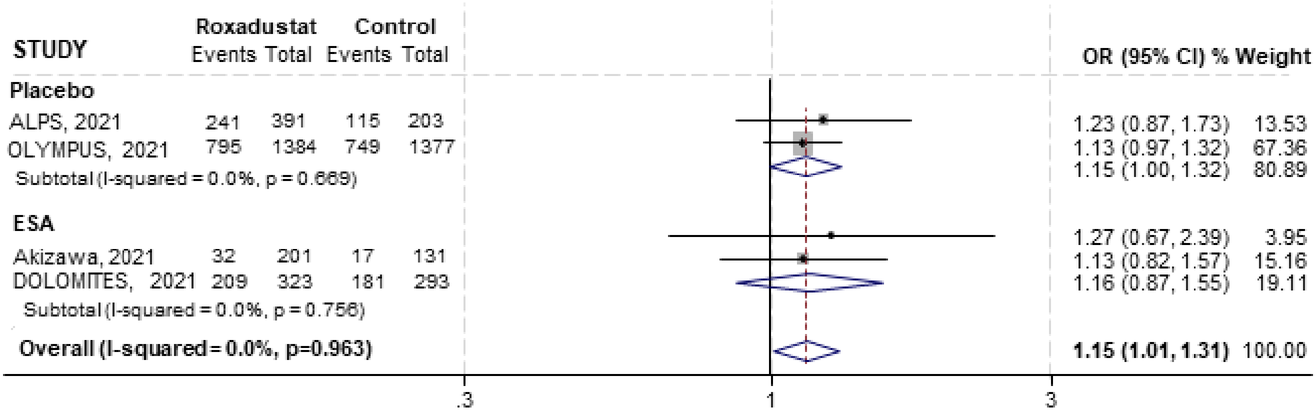

(b) Serious adverse events by treatment duration > 30 weeks in DD patients

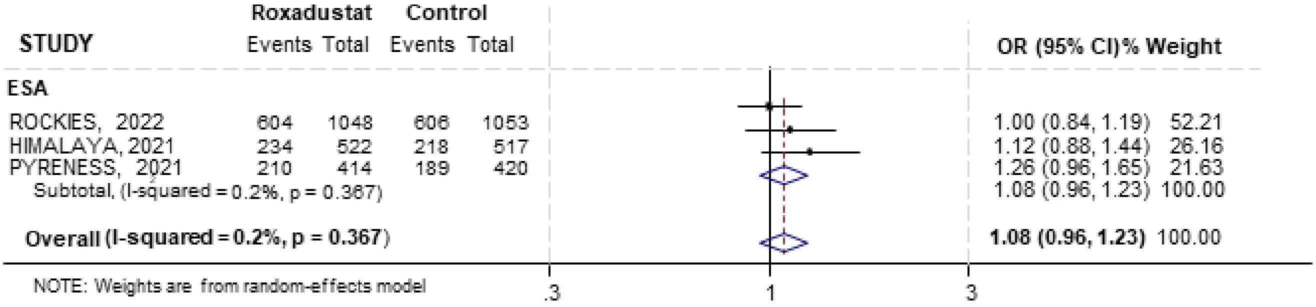

Figure S2. Forest plot of the effect of Roxadustat treatment duration on the incidence of SAEs in anemic patients with CKD, according to the comparator. (a) Patients with serious adverse events analyzed in patients not receiving dialysis with treatment > 30 weeks according to the comparator type, ESA or placebo. **(b)** Patients with serious adverse events analyzed in patients on dialysis with treatment > 30 weeks according to the comparator. All results are presented as odds ratios (ORs) for treatment versus comparator, with their 95% confidence intervals (95% CI).
